# Denture Use and Cognitive Decline in Chinese Older Adults: A Propensity Score Analysis

**DOI:** 10.1093/geroni/igab046.114

**Published:** 2021-12-17

**Authors:** Bei Wu, Xiang Qi

**Affiliations:** 1 New York University, New York, New York, United States; 2 Rory Meyers College of nursing, New York, New York, United States

## Abstract

Using data from the Chinese Longitudinal Healthy Longevity Survey (2011 to 2018), we examined the effect of denture use on cognitive decline (assessed by the Mini-Mental State Examination [MMSE]) among 1,316 cognitively normal older adults with severe tooth loss (≤9 remaining teeth) at baseline. We generated propensity scores for weighted and matched analyses using 18 covariates, classified as socio-demographics, health-related behaviors, health status, and oral health conditions. The results show that non-denture users had worse cognitive decline than denture users. In the kernel-based matched data, the difference in the declined score of cognitive function between denture and non-denture users was 2.25 (95%CI=1.37 to 3.13). In the weighted data, the difference in cognitive function score was 2.14 (95% CI=1.35 to 2.94). Using dentures is beneficial for cognitive health in older adults with severe tooth loss, suggesting that prosthodontic rehabilitation with dentures might have benefits beyond restoring oral functioning.

